# Unprecedented *in Vitro* Antitubercular Activitiy of Manganese(II) Complexes Containing 1,10-Phenanthroline and Dicarboxylate Ligands: Increased Activity, Superior Selectivity, and Lower Toxicity in Comparison to Their Copper(II) Analogs

**DOI:** 10.3389/fmicb.2018.01432

**Published:** 2018-07-02

**Authors:** Pauraic McCarron, Malachy McCann, Michael Devereux, Kevin Kavanagh, Ciaran Skerry, Petros C. Karakousis, Ana C. Aor, Thaís P. Mello, André L. S. Santos, Débora L. Campos, Fernando R. Pavan

**Affiliations:** ^1^Chemistry Department, Maynooth University, National University of Ireland, Maynooth, Ireland; ^2^The Center for Biomimetic and Therapeutic Research, Focas Research Institute, Dublin Institute of Technology, Dublin, Ireland; ^3^Biology Department, Maynooth University, National University of Ireland, Maynooth, Ireland; ^4^Division of Infectious Diseases, Center for Tuberculosis Research, Department of Medicine, Johns Hopkins School of Medicine, Baltimore, MD, United States; ^5^Departamento de Microbiologia Geral, Instituto de Microbiologia Paulo de Góes, Universidade Federal do Rio de Janeiro, Rio de Janeiro, Brazil; ^6^Faculdade de Ciências Farmacêuticas, Universidade Estadual Paulista, Araraquara, São Paulo, Brazil

**Keywords:** *Mycobacterium tuberculosis*, manganese(II), 1, 10-phenanthroline, metal-based complex, antimicrobial agent, *Galleria mellonella*

## Abstract

*Mycobacterium tuberculosis* is the etiologic agent of tuberculosis. The demand for new chemotherapeutics with unique mechanisms of action to treat (multi)resistant strains is an urgent need. The objective of this work was to test the effect of manganese(II) and copper(II) phenanthroline/dicarboxylate complexes against *M. tuberculosis*. The water-soluble Mn(II) complexes, [Mn_2_(oda)(phen)_4_(H_2_O)_2_][Mn_2_(oda)(phen)_4_(oda)_2_]·4H_2_O (**1**) and {[Mn(3,6,9-tdda)(phen)_2_]·3H_2_O·EtOH}n (**3**) (odaH_2_ = octanedioic acid, phen = 1,10-phenanthroline, tddaH_2_ = 3,6,9-trioxaundecanedioic acid), and water-insoluble complexes, [Mn(ph)(phen)(H_2_O)_2_] (**5**), [Mn(ph)(phen)_2_(H_2_O)]·4H_2_O (**6**), [Mn_2_(isoph)_2_(phen)_3_]·4H_2_O (**7**), {[Mn(phen)_2_(H_2_O)_2_]}_2_(isoph)_2_(phen)·12H_2_O (**8**) and [Mn(tereph)(phen)_2_]·5H_2_O (**9**) (phH_2_ = phthalic acid, isophH_2_ = isophthalic acid, terephH_2_ = terephthalic acid), robustly inhibited the viability of *M. tuberculosis* strains, H37Rv and CDC1551. The water-soluble Cu(II) analog of (**1**), [Cu_2_(oda)(phen)_4_](ClO_4_)_2_·2.76H_2_O·EtOH (**2**), was significantly less effective against both strains. Whilst (**3**) retarded H37Rv growth much better than its soluble Cu(II) equivalent, {[Cu(3,6,9-tdda)(phen)_2_]·3H_2_O·EtOH}n (**4**), both were equally efficient against CDC1551. VERO and A549 mammalian cells were highly tolerant to the Mn(II) complexes, culminating in high selectivity index (SI) values. Significantly, *in vivo* studies using *Galleria mellonella* larvae indicated that the metal complexes were minimally toxic to the larvae. The Mn(II) complexes presented low MICs and high SI values (up to 1347), indicating their auspicious potential as novel antitubercular lead agents.

## Introduction

*Mycobacterium tuberculosis* is a pathogenic, acid-alcohol resistant bacillus and is responsible for the highly contagious and potentially fatal disease, tuberculosis (TB) (Ryan et al., 2014). The bacterium has an unusual, impermeable, waxy coating composed mainly of mycolic acids, a feature in part responsible for its inherent resistance to numerous drugs. The infected host is thought to contain populations of *M. tuberculosis* in cavitary lesions, closed caseous lesions, and within macrophages (Bennett, 1994). In cavities, the oxygen level is high, the medium is neutral or slightly alkaline and replication is relatively fast. With the other two populations, the oxygen concentration is lower, the medium is neutral or acidic and multiplication is slower. The disease most commonly involves the lungs and is readily spread via aerosol. However, the infection may also spread to distant sites, such as the brain, kidneys, spleen, liver, and bones (Krishnan et al., 2010). In 2016, there were 9.6 million new cases of TB and 1.5 million deaths (Who.int). Furthermore, there has been an alarming rise in the number of patients presenting with multidrug-resistant (MDR) TB, which is defined by resistance to the two front-line drugs, isoniazid (INH), and rifampicin, and extensively drug-resistant (XDR) TB, which additionally exhibits resistance to two of the most important second-line drug classes (fluoroquinolones and injectable agents). The World Health Organization estimated that *ca*. 4,80,000 people developed MDR-TB in the world in 2014, and that 9.7% of these cases had XDR-TB (Who.int). The treatment for MDR-TB and XDR-TB is costly, toxic, lengthy and less effective than the standard regime, which contributes to medical non-adherence and the emergence of totally drug-resistant strains (TDR-TB). Clearly, in order to effectively treat these highly resistant strains of *M. tuberculosis* there is an urgent need for new drug classes possessing novel mechanisms of action.

Throughout classical antiquity, empirical formulations comprising metal ions have been used for medicinal purposes. However, following the discovery of penicillin and other drugs of biological and synthetic organic origin the clinical use of metallo compounds declined markedly. But within the past 50 years there has been a renaissance in metal-based pharmaceuticals, driven mainly by problems of efficacy and resistance. Some examples of therapeutically important, metal-containing systemic drugs include platinum and arsenic for cancer treatment, samarium for metastatic tumor pain relief, gold as an anti-arthritic, bismuth as a gastrointestinal antimicrobial, antimony and arsenic as anti-parasitics, iron in cardiovascular disease, lithium for bipolar disorder, barium and gadolinium as diagnostic imaging agents, radioactive isotopes of gallium, indium and technetium in tomography, and radiopharmaceuticals containing strontium, yttrium, samarium, and radium (Gielen and Tiekink, 2005; Dabrowiak, 2009; Mjos and Orvig, 2014). Nanoparticulate silver and silver salts are also being applied topically as antibacterial agents in wound and burn treatments (Stobie et al., 2008; Landsdown, 2010).

There are numerous examples where transition metal complexes have been shown to inhibit the growth of *M. tuberculosis in vitro*. Integration of metal ions into the drug structure offers structural diversity, possible access to numerous oxidation states of the metal and the potential of enhancing the efficacy of an established organic drug through its coordination to the metal (Viganor et al., 2015). Metal complexes containing a variety of ligands, such as thiosemicarbazones, quinolones, amines, imines and phenanthrolines, have shown substantial growth inhibition of *M. tuberculosis*. Mechanistic studies have been conducted on metal ligated by the pro-drug INH and some of its derivatives. In particular, the octahedral Fe(II) complex trianion, [Fe(CN)_5_(INH)]^3−^, which returned a MIC value of 0.43 μM (based on Na_3_[Fe(CN)_5_(INH)]·4H_2_O), has been scrutinized in detail (Oliveira et al., 2004). Studies have inferred that the mode of action of INH in blocking the synthesis of *M. tuberculosis* cell wall mycolic acids is linked to the *in situ* formation of coordination complexes with redox-active metal ions like Cu(II) and Fe(II) (Bernardes-Génisson et al., 2013). In the case of [Fe(CN)_5_(INH)]^3−^, it is believed that the Fe(II) center initiates the oxidation of the coordinated INH to form a bioactive species that confers *in vitro* and *in vivo* growth inhibition activity against both INH-sensitive and INH-resistant strains of *M. tuberculosis*. In addition, cytotoxicity studies with [Fe(CN)_5_(INH)]^3−^ against mammalian cancer cells showed IC_50_ values >54 μM, which translated to a credible selectivity index (SI) of >125 (Oliveira et al., 2004). More recently, Poggi et al. (2013) reported MIC values of 2.2 and 0.41 μM for the respective INH-containing Cu(II) and Co(II) complexes, [Cu(INH)(H_2_O)]SO_4_·2H_2_O and [CoCl(INH)_2_(H_2_O)]Cl·2.5H_2_O, against *M. tuberculosis* H37Rv. Both complexes were only very sparingly soluble in water and thought to be more lipophilic than uncoordinated INH. Very encouraging SI values, established using VERO epithelial cells (ATCC CCL81) and macrophage J774A.1 cells (ATCC TIB-67) were obtained for the Cu(II) and Co(II) complexes.

In 1969, Dwyer et al. (1969) published their comprehensive, landmark treatise on the *in vitro* and *in vivo* antibacterial activities of dicationic Mn(II), Fe(II), Co(II), Ni(II), Cu(II), Zn(II), Cd(II), and Ru(II) chelates containing 1,10-phenanthroline (phen), substituted phen (R-phen), 2.2'-bipyridine (bipy), and substituted bipy (R-bipy) ligands. Against *M. tuberculosis* H37Rv the bipy complexes were considerably less potent than their phen analogs. Metal centers chelated by the 5-NO_2_-phen ligand showed the most potent antitubercular activity, with the substitutionally-inert Ru(II) tris chelate being 128-fold less active. Importantly, the bacilli did not develop significant resistance to the 5-NO_2_-phen complexes. However, treatment with the phen complexes did not increase the survival of *M. tuberculosis*-infected mice relative to the untreated rodent (Dwyer et al., 1969). The low *in vivo* activity was attributed to either poor bioavailability at doses safe for the host and/or a failure to access locations where the organism proliferates. More recently, Hoffman and coworkers (Hoffman et al., 2013) prepared mono- and binuclear Co(II) and Cu(II) phen complexes incorporating the water-solubilizing pyrophosphate ligands, formulating as {[Co(phen)_2_]_2_(μ-P_2_O_7_)}, [Co(phen)_2_(H_2_P_2_O_7_)], {[Cu(phen)]_2_(μ-P_2_O_7_)}, and [Cu(phen)(H_2_O)(H_2_P_2_O_7_)]. Mononuclear [Cu(phen)(H_2_O)(H_2_P_2_O_7_)] was the least active against *M. tuberculosis* H37Rv (MIC = 71.53 μM) whilst its mono-Co(II) analog was the most potent (2.05 μM). In addition, all substances were active against MDR ATCC 49967, with the dinuclear complexes, {[M(phen)_2_]_2_(μ-P_2_O_7_)}, showing the greatest activity (MIC values of 2.41 and 2.80 μM, respectively, for Co(II) and Cu(II) derivatives). Additionally, SI values (based on A549 cells) for the Co(II) complexes were much larger than those of their respective Cu(II) counterparts. The complexes resisted efflux mechanisms in mycobacteria and interfered with multiple biochemical pathways. Dholariya et al. (2013) reported the antitubercular activities of Cu(II) complexes ligated by dicoumarol (dicoum) derivatives and phen, formulated as [Cu(dicoum)(phen)(H_2_O)(OH)]·xH_2_O (Devereux et al., 2000). Against *M. tuberculosis* H37Rv, only complexes incorporating hydroxylated (-3-OH) and chlorinated (-4-Cl) dicoumarols showed appreciable activity (MIC_90_ = 4.05 and 64.8 μM, respectively). Patel et al. (2012) tested an array of similar Cu(II) acyl coumarin/phen complexes which displayed only moderate anti*-M. tuberculosis* activity (MIC range 49.5->243 μM). Segura et al. (2014) synthesized Ag(I) thiourea (tu)/phen complexes, [{Ag(phen)(μ-tu)}_2_]X_2_ (X = ${\text{NO}}_{3}^{-}$, CF_3_${\text{SO}}_{3}^{-}$), having MIC values of 11.0 and 14.2 μM (X = ${\text{NO}}_{3}^{-}$ and CF_3_${\text{SO}}_{3}^{-}$, respectively) against H37Rv.

**Figure 1:**
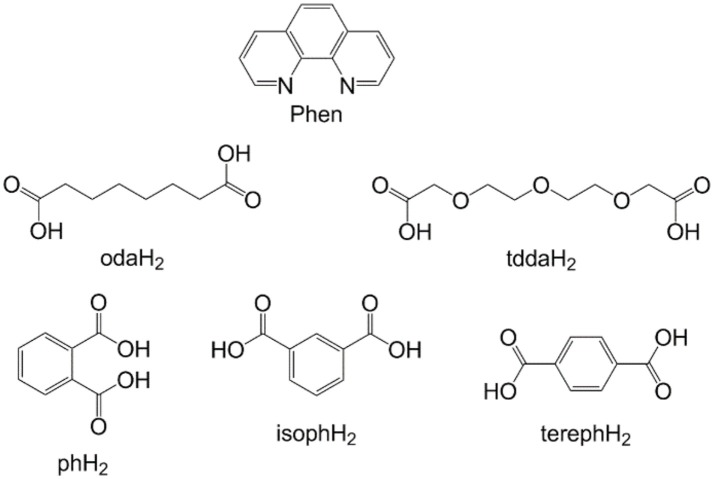
Ligand structures: 1,10-phenanthroline (phen), octanedioic acid (odaH_2_), 3,6,9-trioxaundecanedioic acid (tddaH_2_), phthalic acid (phH_2_), isophthalic acid (isophH_2_), terephthalic acid (terephH_2_).

**Figure 2:**
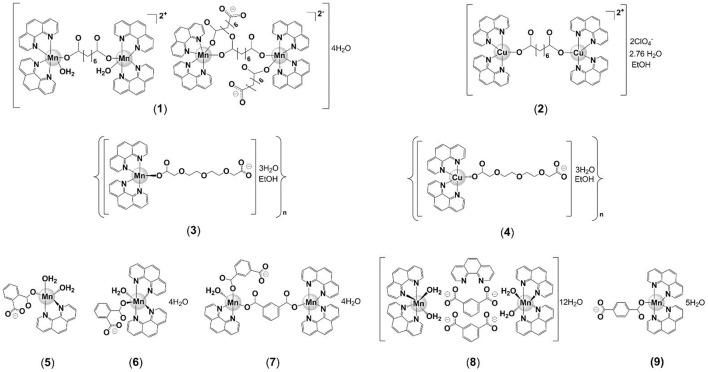
Proposed structures of complexes utilized for anti-tubercular testing: [Mn_2_(oda)(phen)_4_(H_2_O)_2_][Mn_2_(oda)(phen)_4_(oda)_2_]·4H_2_O (**1**), [Cu_2_(oda)(phen)_4_](ClO_4_)_2_·2.76H_2_O·EtOH (**2**), {[Mn(3,6,9-tdda)(phen)_2_]·3H_2_O·EtOH}_n_ (**3**), {[Cu(3,6,9-tdda)(phen)_2_]·3H_2_O·EtOH}_n_ (**4**), [Mn(ph)(phen)(H_2_O)_2_] (**5**), [Mn(ph)(phen)_2_(H_2_O)]·4H_2_O (**6**), [Mn_2_(isoph)_2_(phen)_3_]·4H_2_O (**7**), {[Mn(phen)_2_(H_2_O)_2_]}_2_(isoph)_2_(phen)·12H_2_O (**8**), [Mn(tereph)(phen)_2_]·5H_2_O (**9**).

In the present study, we report the *in vitro* anti-*M. tuberculosis* activity of the water-soluble Mn(II) and Cu(II) phen/dicarboxylate complexes (Figures 1,2), [Mn_2_(oda) (phen)_4_ (H_2_O)_2_][Mn_2_(oda)(phen)_4_(oda)_2_]·4H_2_O(**1**), [Cu_2_(oda)(phen)_4_](ClO4)_2_·2.76H_2_O·EtOH (**2**), {[Mn(3,6,9-tdda)(phen)_2_] ·3H_2_O·EtOH}n (**3**) and {[Cu(3,6,9-tdda)(phen)_2_]·3H_2_O·EtOH}n (**4**) (odaH_2_ = octanedioic acid, tddaH_2_ = 3,6,9-trioxaundecanedioic acid) (Figure 2), and the water-insoluble Mn(II) complexes, [Mn(ph)(phen)(H_2_O)_2_] (**5**), [Mn(ph)(phen)_2_(H_2_O)]·4H_2_O (**6**), [Mn_2_(isoph)_2_(phen)_3_]·4H_2_O (**7**), {[Mn(phen)_2_(H_2_O)_2_]}_2_(isoph)_2_(phen)·12H_2_O (**8**) and [Mn(tereph)(phen)_2_]·5H_2_O (**9**) (phH_2_ = phthalic acid, isophH_2_ = isophthalic acid, terephH_2_ = terephthalic acid) (Figure 2). In addition, we present toxicity profiling data for the complexes, obtained using mammalian VERO (normal kidney) and A549 (adenocarcinomic alveolar) epithelial cells *in vitro* and against *Galleria mellonella* larvae for the *in vivo* systemic toxicity study.

## Materials and methods

### Synthesis of complexes

All the chemicals were purchased from commercial sources and used without further purification. Complexes **1-9** (Devereux et al., 2000; Kellett et al., 2011; Gandra et al., 2017) were prepared as previously reported by our group.

### Mycobacterial strains

The *M. tuberculosis* strains that were utilized for these studies were the well-characterized laboratory reference strain, H37Rv (ATCC 27294) (Cole et al., 1998) and clinically-derived CDC1551 (Manca et al., 1999).

### *In vitro* screening against *M. tuberculosis* H37Rv

The anti-mycobacterial activity of the complexes was determined by the resazurin microtiter assay method (Palomino et al., 2002). Stock solutions of the test complexes were prepared and diluted in Middlebrook 7H9 broth (Difco) supplemented with oleic acid, albumin, dextrose, and catalase (OADC enrichment – BBL/Becton–Dickinson), to obtain the final drug concentration range of 0.09–25 mg/L. INH was dissolved in distilled water and was used as control. A suspension of H37Rv cells was cultured in Middlebrook 7H9 broth supplemented with OADC and 0.05% Tween-80 until an OD_600_ of ≈1.0. The culture was diluted to 5× 10^5^ bacilli per mL and of 100 μL were added to each well of a 96-well microplate together with equal volumes of the complexes. Samples were set up in triplicate. The plates were incubated for 7 days at 37°C. Resazurin (solubilized in water) was added (30 μL of 0.01%). The fluorescence of the wells was read after 24 h with a Cytation 3®. The MIC was defined as the lowest concentration resulting in 90% inhibition of growth of the bacterium.

### *In vitro* screening against *M. tuberculosis* CDC1551

A total of 10^5^ bacilli (OD_600_ = 0.5) were inoculated into separate tubes containing 1 mL of supplemented Middlebrook 7H9 broth lacking Tween. To these cultures increasing (2-fold) concentrations of test compounds were added and the tubes were left standing at 37°C for 14 days. The MIC was defined as the lowest concentration failing to produce a visible pellet.

### Mammalian cell cytotoxicity

A549 cytotoxicity was evaluated using the 3-(4,5-dimethylthiazol-2-yl)-2,5-diphenyl tetrazolium bromide (MTT; Sigma-Aldrich, USA) assay. A549 lung epithelial cells (10^4^) were seeded into tissue culture plates (TPP, Switzerland) and cultured during 24 h in confluence at 37°C in a 5% CO_2_ atmosphere. The wells were then washed twice with DMEM to remove non-adherent cells and the test compounds were added in different concentrations (ranging from 0.0313 to 512 mg/L), followed by incubation the plates for 48 h under the same conditions mentioned above. Subsequently, the cellular viability was evaluated by adding the MTT reagent to each well and by incubating the plates for 3 h, allowing the viable cells containing active mitochondrial dehydrogenases to metabolize the tetrazolium salt into formazan. The formazan crystals were dissolved with DMSO (100 μL) and the absorbance was measured using a Thermomax Molecular Device microplate reader at 450 nm. In parallel, cytotoxicity was also performed on normal epithelial cells VERO (ATCC CCL-81) as previously described by Pavan et al. (2010). The cells were incubated at 37°C in a humidified 5% CO_2_ atmosphere in flasks with a surface area of 12.50 cm^2^ in DMEM medium (10 mL) supplemented with 10% fetal bovine serum, gentamicin sulfate (50 mg/L) and amphotericin B (2 mg/L). The technique consists of collecting the cells using a solution of trypsin/EDTA, centrifugation (2,000 rpm for 5 min), counting the number of cells in a Neubauer chamber and then adjusting the concentration to 3.4 × 10^5^ cells/mL in DMEM. Then, 200 μL of this suspension was deposited in each well of a 96-well microplate to obtain a concentration of 6.8 × 10^4^ cells per well and incubated at 37°C in an atmosphere of 5% CO_2_ for 24 h to allow the cells to attach to the microplate. Dilutions on the test compounds were prepared to obtain concentrations from 500 to 1.95 mg/L. The dilutions were added to the cells after the medium and the non-adherent cells were removed. The cells were then incubated for an additional 24 h. The cytotoxicity of the compounds was determined by adding 30 μL of resazurin and reading on a Synergy H1 (BioTek®) reader after 6 h of incubation using a microplate and excitation and emission filters at wavelengths of 530 nm and 590 nm, respectively. For both cellular systems, the 50% cytotoxic concentration (CC_50_) was defined as the compound concentration which caused a 50% reduction in the number of viable cells. In addition, selectivity index (SI) is calculated as follows: CC_50_ (mammalian cells)/MIC (*M. tuberculosis* cells).

### *In vivo* cytotoxicity

*G. mellonella* larvae in the 6th developmental stage were used to determine the *in vivo* cytotoxicity of the test complexes (Kellett et al., 2011; Desbois and Coote, 2012; McCann et al., 2012). Thirty healthy larvae, each weighing between 0.2 and 0.4 g and with no cuticle discolouration, were used for each experiment. Fresh solutions of the test complexes were prepared immediately prior to testing under sterile conditions. Test compounds (0.05 g) were dissolved in DMSO (1 mL) and added to sterile water (9 mL) to give a stock solution/suspension (5,000 μg/mL). Test solutions/suspensions were prepared from the corresponding stock solution using Millipore water only to dilute to the desired concentration and each compound was screened across the concentration range of 5,000–100 mg/L. Test solutions/suspensions (20 μL) were administered to the larvae by injection directly into the haemocoel through the last pro-leg. The base of the pro-leg can be opened by applying gentle pressure to the sides of the larvae and this aperture will re-seal after removal of the syringe without leaving a scar. Larvae were placed in sterile Petri dishes and incubated at 30°C for 72 h. The survival of the larvae was monitored every 24 h. Death was assessed by the lack of movement in response to stimulus together with discolouration of the cuticle. Three controls were employed in all assays. The first consisted of untouched larvae maintained at the same temperature as the test larvae. The second was larvae with the pro-leg pierced with an inoculation needle but no solution injected. The third control was larvae that were inoculated with 20 μL of sterile water.

## Results

### Anti-mycobacterial activity of metal complexes

*In vitro* growth inhibitory data (MIC values) for the complexes, MnCl_2_·2H_2_O, phen and the first-line anti-mycobacterial agent, INH, against H37Rv and CDC1551 strains of *M. tuberculosis* are displayed in Table 1. Based on μM concentrations, the most active of the Mn(II) complexes against H37Rv were [Mn_2_(oda)(phen)_4_(H_2_O)_2_][Mn_2_(oda)(phen)_4_(oda)_2_]·4H_2_O (**1**) and {[Mn(3,6,9-tdda)(phen)_2_]·3H_2_O·EtOH}n (**3**), which had MIC values comparable to INH (0.44 μM). With the exception of the Mn(II) complex (**3**), all of the metal complexes showed markedly increased inhibitory activity (up to 10-fold in some instances) against strain CDC1551 relative to H37Rv, and many had an MIC value less than that of INH (0.44 μM). Against CDC1551, {[Mn(phen)_2_(H_2_O)_2_]}_2_(isoph)_2_(phen)·12H_2_O (**8**), **1** and [Mn_2_(isoph)_2_(phen)_3_]·4H_2_O (**7**) were the most active (MIC range 0.12–0.18 μM), with an almost 3-fold increase in potency compared to INH. The Cu(II) counterparts of **1** and **3**, i.e., [Cu_2_(oda)(phen)_4_](ClO_4_)_2_·2.76H_2_O·EtOH (**2**) and {[Cu(3,6,9-tdda)(phen)_2_]·3H_2_O·EtOH}n (**4**), were considerably less active against H37Rv, but this disparity was noticeably smaller for the CDC1551 strain, possibly reflecting a degree of specificity by these d^9^ metal complexes.

**Table 1:** *In vitro* MIC values against two strains of *M. tuberculosis* (H37Rv and CDC1551), IC_50_ values for VERO and A549 epithelial cells and calculated SI values for the test complexes and uncoordinated phen.

**Compound**	***M. tuberculosis*** **H37Rv**			***M. tuberculosis*** **CDC1551**			**Cytotoxicity**	
	**MIC_90_ mg/L (μM)**	**SI VERO**	**SI A549**	**MIC_100_ mg/L (μM)**	**SI VERO**	**SI A549**	**IC_50_ mg/L (μM) VERO cells**	**IC_50_ mg/L (μM) A549 cells**
Isoniazid (INH)	0.06 (0.44)	5513	5284	0.06 (0.44)	5513	5284	332.7 (2426)	318.9 (2325)
1,10-phenanthroline (phen)	11.16 (61.93)	1	0.39	3.0 (16.65)	3	1.43	>10 (>55.49)	4.30 (23.9)
[Mn_2_(oda)(phen)_4_(H_2_O)_2_] [Mn_2_(oda)(phen)_4_ (oda)_2_]·4H_2_O (**1**)	1.15 (0.47)	325	445	0.38 (0.15)	1017	1347	375 (152.55)	>512 (>208.3)
[Cu_2_(oda)(phen)_4_](ClO_4_)_2_· 2.76H_2_O·EtOH (**2**)	16.60 (12.68)	0.71	0.06	1.50 (1.15)	8	0.65	11.7 (8.94)	0.98 (74.9)
{[Mn(3,6,9-tdda)(phen)_2_] ·3H_2_O·EtOH}_n_ (**3**)	0.56 (0.76)	112	467	0.75 (1.02)	83	349	62.5 (84.96)	261.67 (355.70)
{[Cu(3,6,9-tdda)(phen)_2_]·3H_2_O·EtOH}_n_ (**4**)	10.03 (13.48)	0.58	0.11	0.75 (1.01)	8	1.41	5.85 (7.86)	1.06 (1.42)
[Mn(ph)(phen)(H_2_O)_2_] (**5**)	0.57 (1.31)	27	411	<0.19 (<0.44)	>82	1234	15.6 (35.84)	234.51 (538.73)
[Mn(ph)(phen)_2_(H_2_O)]·4H_2_O (**6**)	1.42 (2.12)	8	183	0.38 (0.57)	31	682	11.7 (17.47)	259.34 (387.33)
[Mn_2_(isoph)_2_(phen)_3_]·4H_2_O (**7**)	1.56 (1.48)	80	164	<0.19 (<0.18)	>661	1347	125 (118.95)	255.87 (243.50)
{[Mn(phen)_2_(H_2_O)_2_]}_2_(isoph)_2_ (phen)·12H_2_O (**8**)	3.01 (1.85)	16	84	<0.19 (<0.12)	>240	1325	46.9 (28.82)	251.76 (154.70)
[Mn(tereph)(phen)_2_]·5H_2_O (**9**)	3.41 (5.09)	14	74	0.38 (0.57)	123	663	46.9 (70.05)	251.94 (376.28)
MnCl_2_·2H_2_O (Oliveira et al., 2014)	>50 (>154)	<3.7	<6.2	Nt	NA	NA	92.1 (568.7)	153.4 (947.3)

*Included are the MIC values for MnCl_2_·2H_2_O (Oliveira et al., 2014). Selectivity Index (SI) = (CC_50_/MIC); Nt, not tested; NA, not applicable*.

### Activity against mammalian vero and A549 epithelial cells

*In vitro* growth inhibitory data (IC_50_ values) for the compounds against mammalian VERO and A549 epithelial cells are listed in Table 1. Both cell lines were more tolerant of INH and MnCl_2_·2H_2_O than phen and the metal-phen complexes. With the exceptions of phen and {[Cu(3,6,9-tdda)(phen)_2_]·3H_2_O·EtOH}n (**4**), A549 cells were substantially more passive toward the metal complexes than VERO cells. The two Cu(II) complexes, [Cu_2_(oda)(phen)_4_](ClO_4_)_2_·2.76H_2_O·EtOH (**2**) and **4**, were more toxic toward these mammalian cell lines than the seven Mn(II) complexes. Of the Mn(II) test samples, the water-soluble complex double salt, [Mn_2_(oda)(phen)_4_(H_2_O)_2_][Mn_2_(oda)(phen)_4_(oda)_2_]·4H_2_O(**1**), had the smallest impact on the VERO cells, whilst water-insoluble [Mn(ph)(phen)(H_2_O)_2_] (**5**) was the least cytotoxic against A549 cells.

### Activity against *G. mellonella* larvae

*G. mellonella* larvae possess an immune system which is analogous to the human innate immune system and are now commonly employed as a convenient, inexpensive, and less ethically sensitive screening model to ascertain the *in vivo* systemic toxicity of potential drugs, the results of which are comparable to those derived from murine studies (Krishnan et al., 2010; Gandra et al., 2017). Larvae were dosed with varying concentrations of phen and the metal complexes and the percentage of dead larvae was recorded (Table 2). At the highest administered concentration of 0.1 mg (333 mg/kg) of test compound per larvae, 10% of the larvae treated with the metal complexes survived, whilst all of the larvae injected at this concentration with phen died. The Mn(II) complexes, **1**, **3**, and **7**, and the Cu(II) complex, **2**, all showed a marked improvement in survival observed upon decreasing the inoculant concentration to 0.02 mg (67 mg/kg). There were no fatalities when 0.01 mg (33 mg/kg) of the test compounds were dispensed. When assessing the relative toxicity of the test complexes an important consideration is the number of phen ligands each complex contains. From Table 3 it is apparent that the toxicity order, when normalized to the number of phen ligands per complex, is essentially unaltered.

**Table 2:** Percentage mortality of *G. mellonella* larvae 72 h post-injection with various dosages of test compounds.

**Compound**	**Administered amount/% Mortality** μ**g per larvae (mg/kg)**				
		**100 (333.33)**	**40 (133.33)**	**20 (66.67)**	**10 (33.33)**
1,10-phenanthroline (phen)^24^	μmol	0.55	0.22	0.11	0.06
	% Mortality	100%	80%	80%	0%
[Mn_2_(oda)(phen)4(H_2_O)_2_][Mn_2_(oda)(phen)_4_(oda)_2_]·4H_2_O (**1**)^24^	μmol	0.041	0.016	0.008	0.004
	% Mortality	90%	90%	40%	0%
[Cu_2_(oda)(phen)_4_](ClO_4_)_2_·2.76H_2_O·EtOH (**2**)^24^	μmol	0.076	0.030	0.015	0.008
	% Mortality	90%	90%	50%	0%
{[Mn(3,6,9-tdda)(phen)_2_]·3H_2_O·EtOH}_n_ (**3**)	μmol	0.136	0.056	0.028	0.014
	% Mortality	90%	80%	50%	0%
{[Cu(3,6,9-tdda)(phen)_2_]·3H_2_O·EtOH}_n_ (**4**)	μmol	0.134	0.054	0.027	0.013
	% Mortality	90%	90%	80%	0%
[Mn(ph)(phen)(H_2_O)_2_] (**5**)	μmol	0.230	0.092	0.046	0.023
	% Mortality	90%	90%	80%	0%
[Mn(ph)(phen)_2_(H_2_O)]·4H_2_O (**6**)	μmol	0.149	0.060	0.030	0.015
	% Mortality	90%	90%	80%	0%
[Mn_2_(isoph)_2_(phen)_3_]·4H_2_O (**7**)	μmol	0.095	0.040	0.020	0.010
	% Mortality	90%	80%	60%	0%
{[Mn(phen)_2_(H_2_O)_2_]}_2_(isoph)_2_(phen)·12H_2_O (**8**)	μmol	0.061	0.025	0.012	0.006
	% Mortality	90%	90%	80%	0%
[Mn(tereph)(phen)_2_]·5H_2_O (**9**)	μmol	0.149	0.060	0.030	0.015
	% Mortality	90%	90%	80%	0%

**Table 3:** Toxicity ordering of the compounds against *G. mellonella* larvae when the results from Table 2 are normalized with respect to phen content.

	***In vivo G. mellonella* tolerance order in μmol**	**No. of phens**		***In vivo G. mellonella* tolerance order in μmol (Normalized against no. of phens)**	**No. of phens**	
1st	[Mn_2_(oda)(phen)_4_(H_2_O)_2_][Mn_2_(oda)(phen)_4_(oda)_2_]· 4H_2_O (**1**)	8	1st	[Mn_2_(oda)(phen)_4_(H_2_O)_2_][Mn_2_(oda)(phen)_4_(oda)_2_]· 4H_2_O (**1**)	8	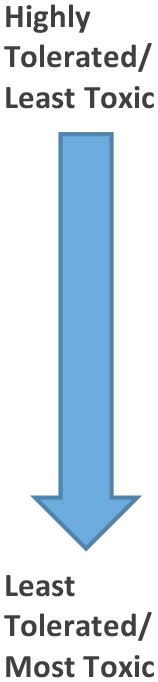
2nd	{[Mn(3,6,9-tdda)(phen)_2_]·3H_2_O·EtOH}_n_ (**3**)	2	2nd	{[Mn(3,6,9-tdda)(phen)_2_]·3H_2_O·EtOH}_n_ (**3**)	2	
3rd	[Cu_2_(oda)(phen)_4_](ClO_4_)_2_·2.76H_2_O·EtOH (**2**)	4	2nd	[Cu_2_(oda)(phen)_4_](ClO_4_)_2_·2.76H_2_O·EtOH (**2**)	4	
4th	[Mn_2_(isoph)_2_(phen)_3_]·4H_2_O (**7**)	3	3rd	[Mn_2_(isoph)_2_(phen)_3_]·4H_2_O (**7**)	3	
5th	1,10-phenanthroline (phen)	1	4th	1,10-phenanthroline (phen)	1	
6th	[Mn(ph)(phen)(H_2_O)_2_] (**5**)	1	5th	[Mn(ph)(phen)_2_(H_2_O)]·4H_2_O (**6**)	2	
7th	[Mn(ph)(phen)_2_(H_2_O)]·4H_2_O (**6**)	2	5th	{[Mn(phen)_2_(H_2_O)_2_]}_2_(isoph)_2_(phen)·12H_2_O (**8**)	5	
7th	[Mn(tereph)(phen)_2_]·5H_2_O (**9**)	2	5th	[Mn(tereph)(phen)_2_]·5H_2_O (**9**)	2	
8th	{[Cu(3,6,9-tdda)(phen)_2_]·3H_2_O·EtOH}_n_ (**4**)	2	6th	{[Cu(3,6,9-tdda)(phen)_2_]·3H_2_O·EtOH}_n_ (**4**)	2	
9th	{[Mn(phen)_2_(H_2_O)_2_]}_2_(isoph)_2_(phen)·12H_2_O (**8**)	5	6th	[Mn(ph)(phen)(H_2_O)_2_] (**5**)	1	

## Discussion

Although the two mycobacterial strains, H37Rv and CDC1551, are equally virulent (Manca et al., 1999) the latter strain is known to induce a more rapid and robust host response in a mouse-infected model. The seven Mn(II) complexes were more active against the H37Rv strain than the two Cu(II) samples, but this inequality was less pronounced against the CDC1551 strain, probably due to difference at media culture. Previous studies involving the fungal pathogen, *Candida albicans*, revealed that the dicarboxylic acids alone were not bioactive (Devereux et al., 2000). This finding, coupled with the fact that the current Mn(II) phen/dicarboxylate complexes are 30–328 times more active against H37Rv than MnCl_2_·2H_2_O and also show 12–132 times greater activity than phen alone (Table 1), suggests the existence of a positive synergism between the Mn(II) dication and its original phen/dicarboxylate ligand set.

The low MIC values for [Mn_2_(oda)(phen)_4_(H_2_O)_2_] [Mn_2_(oda)(phen)_4_(oda)_2_]·4H_2_O (**1**) and {[Mn(3,6,9-tdda)(phen)_2_]·3H_2_O·EtOH}n (**3**) against H37Rv translated to strikingly large selectivity index (SI) values as shown in Table 1 (325/445 and 112/467, respectively, for VERO/A549 cells). Furthermore, the 3-fold increase in activity against CDC1551 over H37Rv for **1** elevated the SI values to 1017/1347. As the Cu(II) complexes, **2** and **4**, were relatively more toxic than the Mn(II) complexes toward the two types of mammalian cells, this severely reduced the SI values of the Cu(II) complexes. The highly cytotoxic nature of **2** and **4** toward A549 cells parallels our previous findings for Cu(II)phen/diacid complexes against cancer cells (Kellett et al., 2011). The isophthalate/phen complexes, [Mn_2_(isoph)_2_(phen)_3_]·4H_2_O (**7**) and {[Mn(phen)_2_(H_2_O)_2_]}_2_(isoph)_2_(phen)·12H_2_O (**8**), showed highly favorable SI values for CDC1551 when referenced against VERO/A549 cells (>661/1347 and >240/1325, respectively), and this is mainly attributable to their very low MIC_100_ values (<0.18, <0.12 μM) against the CDC1551 strain.

Dwyer et al. (1969) reported that the Mn(II), Cu(II), Zn(II) and Cd(II) phen dicationic complexes, [M(phen)_2_](CH_3_CO_2_)_2_ and [M(R-phen)_2_](CH_3_CO_2_)_2_, all had similar activities against H37Rv with MIC values ranging from approximately 30 μM for [M(phen)_2_]^2+^ to 0.1 μM for [M(5-NO_2_-phen)_2_]^2+^. This uniformity in activity between Mn(II) and Cu(II) contrasts somewhat to our findings, which clearly show that in the case of the phen/oda and phen/tdda ligand combinations the Mn(II) complexes were 27- and 18-fold more active, respectively, against H37Rv than their Cu(II) analogs. Against CDC1551, the difference between the Mn(II) phen/oda and Cu(II) complexes was less marked (10-fold), whilst the two phen/tdda samples showed the same activity. It is primarily the high tolerance of the mammalian cells toward the Mn(II) complexes, in contrast to their Cu(II) analogs, that accounts for the remarkably high SI values of the Mn(II) complexes. Also of relevance to the present work is a recent publication (Oliveira et al., 2014) describing the activity of a collection of water-insoluble, octahedral Mn(II) complexes of formula, [Mn(atc-R)_2_] (atc-R = tridentate 2-acetylpyridine-N(4)-R-thiosemicarbazone anion), against *M. tuberculosis* H37Rv. MIC values, which were dependent on the nature of the pendant R group, ranged from 50.69 to 1.31 μM, with a corresponding SI range (measured against VERO cells) of 5.3->641 (for [Mn(atc-Me)_2_] to [Mn(atc-Ph)_2_], respectively). On comparing the excellent and somewhat similar SI values of hydrophobic [Mn(atc-Ph)_2_] with relatively hydrophilic [Mn_2_(oda)(phen)_4_(H_2_O)_2_][Mn_2_(oda)(phen)_4_(oda)_2_]·4H_2_O (**1**), it is the very high IC_50_ value for [Mn(atc-Ph)_2_] that is the dominant feature, whilst for **1** the major contributing factor is the extremely low MIC value.

Whilst relatively small quantities of transition metal ions (primarily Mn, Fe, Co, Ni, Cu, Zn) are essential micronutrients for sustaining microbial growth and homeostasis (Braymer and Giedroc, 2014; Neyrolles et al., 2015), exposure to high concentrations can be devastating as they can bind to and disable important biomolecules and/or promote oxidative stress through Fenton chemistry. It is important to note that Mn(II) and Cu(II) complexes are kinetically labile, meaning that they can rapidly exchange their original ligands (phen and dicarboxylate in the present cases) for other donor ligands present in a biological milieu which includes the bacterium itself. Thus, it is anticipated that a dynamic equilibrium is rapidly established between the original M(II)-phen/dicarboxylate and the newly formed various M(II)-bioligand complexes.

There are several potential explanations for the superior activity of the Mn(II) phen/dicarboxylates **1** and **3** against *M. tuberculosis* H37Rv relative to their Cu(II) equivalents, **2** and **4**. Various metal dication transporter proteins have been identified for *M. tuberculosis*, which includes CTR1 for Cu(II), [(Manca et al., 1999) multimetal Mramp (Mn(II), Fe(II), Cu(II) and Zn(II)] (Agranoff et al., 1999) and a fleet of P_1B_-ATPases for Mn(II), Cu(II) and some other metals (Padilla-Benavides et al., 2013). It is known that Cu(II) must be reduced to Cu(I), possibly by membrane associated copper reductases (Hassett and Kosman, 1995), before being recruited by the Cu(I) importer protein, CTR1. On the other hand, metallothioneins present in the bacterial cytosol prevent copper overload and toxicity by sequestering surplus copper ions (Wang et al., 2011). In addition, the copper transporter protein, ATP7A, can translocate to the plasma membrane and pump excess copper out of the cell (Petris and Mercer, 1999). The outer membrane channel protein, Rv1698, of *M. tuberculosis* is also reported to efflux excess Cu(II) from the mycobacterial cell envelope, a process which is necessary for its survival (Wolschendorf et al., 2011). Likewise, the membrane protein, MctB, reduces intracellular copper levels and is required for full *M. tuberculosis* copper resistance and virulence in mice and guinea pigs (Wolschendorf et al., 2011). It may be an inability to reduce administered Cu(II) to Cu(I) and/or sequestration and efflux that is managing to buffer the amount of Cu(II) to the relatively non-hazardous levels observed for the current Cu(II) complexes against H37Rv in comparison to their Mn(II) analogs.

Recent research has shown that *M. tuberculosis* may be highly susceptible to specific types of reactive oxygen species (ROS) and reactive nitrogen species (RNS) (Cirillo et al., 2009; Voskuil et al., 2011; Roca and Ramakrishnan, 2013; Vilcheze et al., 2013), and the bacteria releases a defensive brigade of enzymes/proteins to counteract the oxidative and nitrosative onslaught by mammalian host cells (Kim et al., 2012). Whilst nitric oxide (NO) has a bacteriostatic effect on *M. tuberculosis* H_2_O_2_ is not bacteriostatic at concentrations below 50 mM, but above this concentration the peroxide is bactericidal (Voskuil et al., 2011). Thus, it would appear that *M. tuberculosis* cells are not equally or universally susceptibility to ROS or RNS and that this may help explain the superior growth inhibitory effects exhibited by the Mn(II)-based test complexes over the Cu(II) complexes, especially against the H37Rv strain. Complex **1** is an avid generator of intracellular ROS (Kellett et al., 2011) and its strong anti-mycobacterial activity may be due to the type and quantity of free radicals or ROS/RNS (superoxide ${\text{O}}_{2}\cdot^{-}$, hydroxyl radical ·OH, ·NO) that the Mn(II) complexes produce. It is conceivable that **1** is generating higher levels of ${\text{O}}_{2}\cdot^{-}$, ·NO and H_2_O_2_ than its Cu(II)-based analog, **2**. In addition, the production of extremely destructive ·OH radicals by **1** may also account for its high anti-mycobacterial activity, as well as that of all of the test Mn(II) complexes. If this is indeed the mode of action of these Mn(II) complexes, then they could possibly overwhelm any strain with acquired resistance to INH. INH is a prodrug and is oxidized by a bacterial catalase-peroxidase enzyme (KatG) present in *M. tuberculosis*, forming an isonicotinic acyl moiety (either an acyl radical or acyl anion). The acyl moiety forms a strong covalent bond to the nicotinamide ring of the nicotinamide adenine dinucleotide cation (NAD^+^), and this acyl-NAD entity docks into the active site of the enoyl-acyl carrier protein reductase, InhA, the enzyme which mediates fatty acid synthesis (Dessen et al., 1995; Zabinski and Blanchard, 1997; Rozwarski et al., 1998; Oliveira et al., 2014). Fatty acids are required for the subsequent production of mycolic acid, which is a key component of the mycobacterial cell wall. Thus, INH indirectly blocks bacterial cell wall construction, leading to the demise of the organism. INH resistance is frequently associated with KatG structural gene alterations, resulting in KatG mutant enzymes with reduced ability to form activated INH compounds (Jagielski et al., 2014). Both KatG and Mn complexes/ions are able to oxidize INH and form the active isonicotinoyl–NAD adduct (Magliozzo and Marcinkeviciene, 1997; Oliveira et al., 2014; Viganor et al., 2015). Also of note is that *M. smegmatis*, a closely related but non-pathogenic bacterium, contains a variant of KatG which has been shown to require Mn(II) ions for activation of INH, possibly via oxidation of Mn(II) to Mn(III) which in turn oxidizes INH (Magliozzo and Marcinkeviciene, 1997). Conversely, Mn(II) ions are not essential for *M. tuberculosis* KatG-mediated oxidative activation of INH although the addition of exogenous Mn(II) ions has been shown to enhance the activation of INH by wild-type and various mutants of *M. tuberculosis* KatG (Lei et al., 2000; Wei et al., 2003). Interestingly, INH-resistant clinical isolates of *M. tuberculosis* have a high incidence of a mutant variant of the KatG enzyme, namely KatG S315, in which the replacement of a serine residue at position 315 in the catalytic domain results in the inability to oxidize INH to isonicotinic acid (Wei et al., 2003; Jagielski et al., 2014). The ability to oxidize INH can be restored to wild-type KatG S315 mutants and other KatG variants (obtained via site directed mutagenesis) by the addition of Mn(II) ions (Lei et al., 2000; Wei et al., 2003). *M. smegmatis* cells, unlike most strains of *M. tuberculosis*, are intrinsically highly resistant to INH (MIC >30 mg/mL; >218.76 mM), a feature which may be due, in part, to the low levels of Mn(II) ions present in *M. smegmatis* cells *in vivo* (Wei et al., 2003). Therefore, co-administration of INH with the current Mn(II)-based complexes could result in the metal complex acting as an alternative oxidant, mimicking the activity of KatG and thus providing a non-enzymatic oxidation and consequent activation of INH, whilst also independently expressing its ROS-mediated growth inhibitory effect on the bacteria. Investigating a possible positive synergism between the Mn(II) complexes and INH will be the focus of a future investigation by our research network.

Encouragingly, all of the metal complexes appear to be well-tolerated, *in vivo*, by *G. mellonella* larvae (Tables 2 3). These data suggest that the relative *in vivo* toxicity profile of the metal complexes is not dependent on the number of coordinated phen ligands present per complex. In addition, regarding the nuclearity (mononuclear, binuclear, tetranuclear) of the metal complexes, there does not appear to be an increase in toxicity upon increasing the metal content of the complex. For example, the tetra-Mn(II) complex **1** is much less toxic toward *G. mellonella* than the mono-Mn(II) complex **9**. Similarly, the di-Cu(II) species **2** is better tolerated than mono-Cu(II) **4**. Although Mn(II) and Cu(II) complexes are inherently liable, comparisons based on phen and metal content of the complexes suggest that it is the complex as a whole, rather than the individual components of the complexes, that are responsible for the observed effects on the larvae.

## Conclusion

In conclusion, Mn(II) phen/dicarboxylate complexes, which can be synthesized efficiently, utilizing economical and readily available starting materials, offer realistic promise as effective, selective and safe lead candidates in the search for new drugs for the treatment of TB.

## Author contributions

PM, MM, MD, KK, CS, PK, AA, TM, AS, DC, and FP conceived and designed the study. PM, MM, AS, and FP analyzed the data and wrote the manuscript. All authors approved of the manuscript.

### Conflict of interest statement

The authors declare that the research was conducted in the absence of any commercial or financial relationships that could be construed as a potential conflict of interest.
